# Structural MRI profiles and tau correlates of atrophy in autopsy-confirmed CTE

**DOI:** 10.1186/s13195-021-00928-y

**Published:** 2021-12-07

**Authors:** Michael L. Alosco, Asim Z. Mian, Karen Buch, Chad W. Farris, Madeline Uretsky, Yorghos Tripodis, Zachary Baucom, Brett Martin, Joseph Palmisano, Christian Puzo, Ting Fang Alvin Ang, Prajakta Joshi, Lee E. Goldstein, Rhoda Au, Douglas I. Katz, Brigid Dwyer, Daniel H. Daneshvar, Christopher Nowinski, Robert C. Cantu, Neil W. Kowall, Bertrand Russell Huber, Victor E. Alvarez, Robert A. Stern, Thor D. Stein, Ronald J. Killiany, Ann C. McKee, Jesse Mez

**Affiliations:** 1 Boston University Alzheimer’s Disease Research Center and CTE Center, Department of Neurology, Boston University School of Medicine, 72 E Concord Street, Suite B7800, Boston, MA 02118 USA; 2 Department of Radiology, Boston University School of Medicine, Boston, USA; 3 Department of Radiology, Massachusetts General Hospital, Boston, USA; 4 Department of Biostatistics, Boston University School of Public Health, Boston, USA; 5 Biostatistics and Epidemiology Data Analytics Center, Boston University School of Public Health, Boston, USA; 6 Framingham Heart Study, Boston University School of Medicine, 72 E Concord Street, Suite B7800, Boston, MA 02118 USA; 7 Department of Pathology and Laboratory Medicine, Boston University School of Medicine, Boston, USA; 8 Department of Psychiatry, Boston University School of Medicine, Boston, USA; 9 Departments of Biomedical, Electrical & Computer Engineering, Boston University College of Engineering, Boston, USA; 10 Department of Anatomy and Neurobiology, Boston University School of Medicine, Boston, USA; 11 Department of Epidemiology, Boston University School of Public Health, Boston, USA; 12 Braintree Rehabilitation Hospital, Braintree, MA USA; 13 Concussion Legacy Foundation, Boston, MA USA; 14 Department of Neurosurgery, Boston University School of Medicine, Boston, USA; 15 Department of Neurosurgery, Emerson Hospital, Concord, USA; 16 US Department of Veteran Affairs, VA Boston Healthcare System, Boston, USA; 17 National Center for PTSD, VA Boston Healthcare, Boston, USA; 18 Department of Veterans Affairs Medical Center, Bedford, MA USA; 19 Center for Biomedical Imaging, Boston University School of Medicine, Boston, USA

**Keywords:** Atrophy, Chronic traumatic encephalopathy, Magnetic resonance imaging, Neurodegeneration, Tau

## Abstract

**Background:**

Chronic traumatic encephalopathy (CTE), a neurodegenerative tauopathy, cannot currently be diagnosed during life. Atrophy patterns on magnetic resonance imaging could be an effective in vivo biomarker of CTE, but have not been characterized. Mechanisms of neurodegeneration in CTE are unknown. Here, we characterized macrostructural magnetic resonance imaging features of brain donors with autopsy-confirmed CTE. The association between hyperphosphorylated tau (p-tau) and atrophy on magnetic resonance imaging was examined.

**Methods:**

Magnetic resonance imaging scans were obtained by medical record requests for 55 deceased symptomatic men with autopsy-confirmed CTE and 31 men (*n* = 11 deceased) with normal cognition at the time of the scan, all >60 years Three neuroradiologists visually rated regional atrophy and microvascular disease (0 [none]–4 [severe]), microbleeds, and cavum septum pellucidum presence. Neuropathologists rated tau severity and atrophy at autopsy using semi-quantitative scales.

**Results:**

Compared to unimpaired males, donors with CTE (45/55=stage III/IV) had greater atrophy of the orbital-frontal (mean diff.=1.29), dorsolateral frontal (mean diff.=1.31), superior frontal (mean diff.=1.05), anterior temporal (mean diff.=1.57), and medial temporal lobes (mean diff.=1.60), and larger lateral (mean diff.=1.72) and third (mean diff.=0.80) ventricles, controlling for age at scan (ps<0.05). There were no effects for posterior atrophy or microvascular disease. Donors with CTE had increased odds of a cavum septum pellucidum (OR = 6.7, *p* < 0.05). Among donors with CTE, greater tau severity across 14 regions corresponded to greater atrophy on magnetic resonance imaging (beta = 0.68, *p* < 0.01).

**Conclusions:**

These findings support frontal-temporal atrophy as a magnetic resonance imaging finding of CTE and show p-tau accumulation is associated with atrophy in CTE.

## Background

Chronic traumatic encephalopathy (CTE) is a neurodegenerative disease associated with exposure to repetitive head impacts (RHI), such as those from contact sport participation [1–5]. Currently, CTE can only be diagnosed at autopsy using neuropathological diagnostic criteria [6]. The pathognomonic lesion of CTE includes hyperphosphorylated tau (p-tau) in neurons, with or without astrocytes, around small blood vessels at the depths of the cerebral sulci [3, 6, 7]. Four pathological stages of CTE have been defined, ranging from stage I (mild) to stage IV (severe) [3, 4, 6, 7]. In stage I CTE, 1 or 2 isolated foci of p-tau neurofibrillary tangles are found, most frequently in the frontal cortex. In stage II CTE, p-tau lesions and superficial tangles spread to adjacent temporal cortices. In stage III CTE, tangles are diffusely distributed in medial temporal lobe (MTL) structures. In stage IV CTE, perivascular p-tau lesions and tangles are distributed throughout the cerebral cortex, with pronounced neurofibrillary degeneration of the MTL. Neuronal loss and gliosis are prominent in the frontal and temporal cortices. Gross features include progressive cerebral, MTL, and anterior diencephalic atrophy. Frontal and temporal lobe atrophy are initial and most prominent. There is marked MTL atrophy by stage III CTE [3, 6]. Other common gross features include a cavum septum pellucidum (CSP) and corpus callosum thinning.

Our understanding of the clinical presentation of CTE has improved [2–4], but still lags behind other tauopathies. At this time, CTE cannot be diagnosed accurately in life, partially due to the lack of validated in vivo biomarkers that can detect CTE pathology and differentiate it from other neurological disorders, like Alzheimer’s disease (AD). Tau positron emission tomography imaging [8, 9] and cerebrospinal fluid protein analysis [10] hold promise for the detection of CTE p-tau pathology in the central nervous system, but these are still under investigation and may lack feasibility due to high costs and/or perception of invasiveness.

Structural magnetic resonance imaging (MRI) is an integral component of the clinical evaluation of neurodegenerative diseases. Atrophy patterns on MRI are non-specific biomarkers of neurodegeneration, but are used to support diagnosis and monitoring of neurodegenerative diseases, like AD and frontotemporal dementia (FTD). Atrophy rates serve as outcomes for large-scale multi-center clinical trials of disease-modifying therapies. It is clinically essential that structural MRI signature(s) of CTE are discerned. Preliminary studies on structural MRI patterns among living individuals at risk for CTE (e.g., former elite football players, fighters) have shown reduced volume of the frontal and temporal lobes [9] and MTL [9, 11–14], greater white matter abnormalities [15, 16], and higher rates of a CSP [17, 18]. These studies lacked gold-standard assessment for CTE pathology and thus could not definitively characterize the specific in vivo structural MRI patterns of CTE.

The causes of atrophy or neurodegeneration in CTE are also poorly understood. In AD and related dementias, p-tau is a driver of atrophy and clinical decline [19–21]. In CTE, it is hypothesized that p-tau aggregates are precipitated by exposure to RHI and accumulate and spread with age [7], compromising neuronal integrity and triggering widespread cell death. The contribution of p-tau to neurodegeneration in CTE has not been empirically tested.

Clinical-pathological correlation studies are essential to establish biomarker patterns of CTE and to identify the mechanisms of disease pathogenesis and neurodegeneration. We examined the MRI patterns of CTE by comparing visually rated macrostructural features (using established visual rating scales) on antemortem MRIs in brain donors with autopsy-confirmed CTE, all of whom were reported to be symptomatic, and participants with normal cognition (NC) (deceased and living, only six of whom donated their brain for autopsy examination). To determine if CTE p-tau pathology is a driver of neurodegeneration, like in AD [19, 21], we tested associations between p-tau severity in donors with CTE and atrophy on in vivo MRI assessments. An initial step in biomarker development is to determine whether the target biomarker can detect disease presence. For this reason, we included participants with NC as a comparison group, acknowledging that comparison with other neurodegenerative diseases would be an important next step to establish biomarker specificity.

## Methods

### Study design

The sample included 55 brain donors with neuropathologically confirmed CTE and 31 participants with NC (combination of deceased and living, further details in the “Clinical research diagnosis of NC” section). Those with neuropathologically confirmed CTE were from the Veteran’s Affairs-Boston University-Concussion Legacy Foundation brain bank and were part of the Understanding Neurologic Injury in Traumatic Encephalopathy (UNITE) study. Participants with NC were from the Boston University Alzheimer’s Disease Research Center (BU ADRC) Clinical Core (*n* = 19) or the Framingham Heart Study (FHS, *n* = 12). Procedures for all studies were approved by the BU Medical Campus and/or the Bedford VA Hospital Institutional Review Board. All informants of brain donors and participants from the BU ADRC and FHS provided written informed consent. Methodological descriptions of the UNITE study [4, 22], the BU ADRC [23, 24], and the FHS [25–27] have been published elsewhere. The following is an overview of each study:

#### UNITE

The objective of UNITE is to characterize the neuropathology and clinical-pathological correlates of CTE and other long-term consequences of RHI. It is made up of brain donors (*N* = 863 at the time of this study) who have a history of RHI (e.g., from contact sport play, military service, physical violence) regardless of whether symptoms were present during life. Next of kin contact the BU CTE Center to arrange brain donation near the time of death or following death. Other brain donors are referred by medical examiners, recruited by the Concussion Legacy Foundation, or agree to donation during life. Brain donors are excluded for prolonged post-mortem interval (i.e., >72 h) or poor tissue quality. In addition to neuropathological examinations, retrospective clinical evaluations (blinded to the neuropathological results) are performed using online questionnaires and structured and semi-structured telephone interviews between researchers and informants of brain donors to ascertain demographic, athletic, clinical, military, traumatic brain injury, and RHI exposure characteristics. Through these methods, a detailed chronology of the donors cognitive, behavioral, mood, motor, and functional symptoms are ascertained. This data is reviewed by a multidisciplinary consensus panel to determine if antemortem dementia was present.

#### BU ADRC

This center is one of ~30 centers funded by the National Institute on Aging that provides data to the National Alzheimer’s Coordinating Center to facilitate collaborative research on AD and related dementias. It longitudinally follows approximately 400 older adults with and without cognitive impairment. Inclusion criteria include English speaking older adults who have adequate hearing and visual acuity. Participants are excluded for a history of a serious mental illness (e.g., bipolar disorder, schizophrenia), confounding neurological disorders (e.g., brain tumor, multiple sclerosis), or medical conditions that preclude study participation. Participants complete annual clinical and medical history interviews, neurological examinations, neuropsychological testing, and measures of functional independence, among other procedures.

#### FHS

This is a longitudinal community-based study that began in 1948. It involves serial examinations of the Original 1948 cohort, as well as of the original cohort participants’ children (i.e., Generation 2, “Offspring Cohort”) and grandchildren (i.e., Generation 3, “Third Generation Cohort”). Recruitment of the OMNI1 Cohort began in 1994 and was aimed to improve representation of the evolving racial and ethnic make-up of the Framingham, Massachusetts community. Of the 12 FHS participants in this study, 9 were from Generation 2, 2 were from Generation 1, and 1 was from the OMNI1 Cohort. Participants complete detailed medical and physical examinations, laboratory tests, neurological evaluations, and neuropsychological testing, among other procedures, every 2 years for the Generation 1 Cohort and approximately every 4 years for the Generation 2 and OMNI1 Cohorts.

### Brain donor and participant selection

**Fig. 1:**
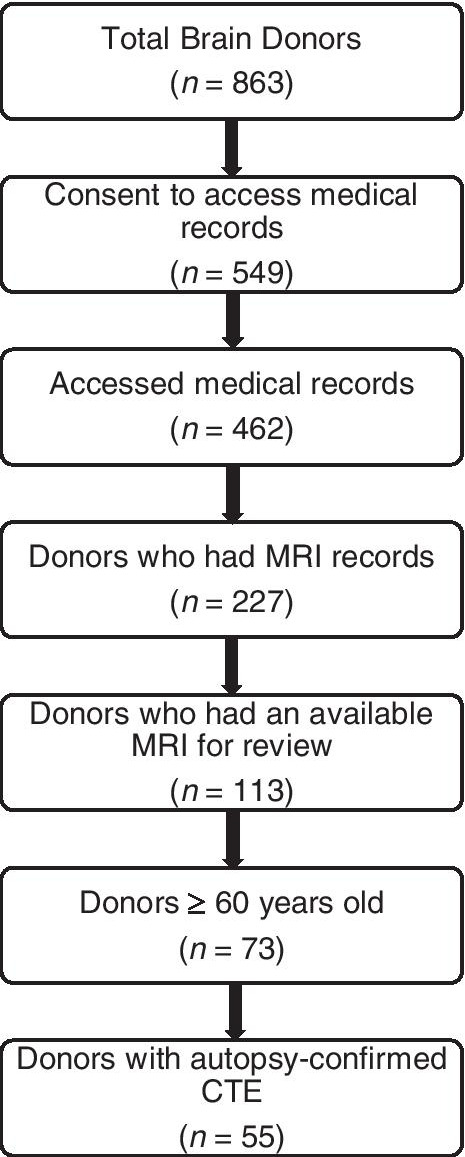
Sample derivation of brain donors with autopsy-confirmed CTE

Refer to Fig. 1 for a flowchart of sample derivation of brain donors. Brain donors with CTE were not followed in life as part of a research study and did not undergo a research-grade MRI. Therefore, the sample was restricted to donors with CTE who underwent an antemortem MRI that could be obtained through medical record request. Only men met these criteria. If more than one clinical MRI was obtained, the most recent was used. The BU ADRC and the FHS were drawn from to obtain a comparison group of men with NC. The present sample was restricted to age 60 or older to facilitate similar age distributions across groups. To mirror procedures utilized for the brain donors with CTE, we only included participants with NC who had available MRIs obtained through medical record request. We did not draw on the research grade MRIs that participants completed during life as part of their participation in the BU ADRC and FHS because it would have resulted in a comparison group with substantially better quality MRIs that could influence visual ratings. Due to varied locations of care and policies for obtaining medical records, obtaining MRIs from medical records significantly reduced the available pool of participants with NC from the BU ADRC and FHS to be included in this study. In addition, the BU ADRC and FHS have substantial representation of older women and thus our age and sex restrictions further limited the pool of eligible NC. After the aforementioned criteria were applied, our initial analyses of the data showed sufficient statistical power to detect group differences, which justified our final analytic sample of 55 brain donors with CTE and 31 participants with NC.

### Diagnostic procedures

#### Neuropathological evaluation

Neuropathological evaluation occurred blinded to clinical data and was reviewed by four neuropathologists (BH, TS, ACM, VA); any discrepancies in the neuropathological diagnoses were resolved by discussion and consensus of the group. Methods for pathological processing and evaluation have been published elsewhere [3, 4, 6, 7, 22, 28] and follow established procedures [29, 30]. Brain weight and macroscopic features were recorded during initial processing. Twenty-two sections of paraffin-embedded tissue were stained for Luxol fast blue, hematoxylin and eosin, Bielschowsky’s silver, p-tau (AT8), alpha-synuclein, beta amyloid (Aß), and phosphorylated TDP-43 using methods described previously [31]. The neuropathological diagnosis of CTE was made using criteria defined by the National Institutes of Neurological Disease and Stroke/National Institute of Brain Imaging and Behavior (NINDS/NIBIB) consensus panel [6, 32]. According to the 2016 NINDS/NIBIB panel, the pathognomonic lesion of CTE was defined as “an accumulation of abnormal hyperphosphorylated tau (p-tau) in neurons and astroglia distributed around small blood vessels at the depths of cortical sulci and in an irregular pattern.” It has been clarified that the neuropathological diagnosis of CTE requires the presence of at least one pathognomonic p-tau lesion in the cortex and astrocytic perivascular p-tau lesions are non-diagnostic [7, 32, 33]. Supportive diagnostic features included neurofibrillary tangles in superficial cortical layers (layers II/III) of the cerebral cortex and pretangles, tangles, or dendritic dystrophy in CA2 and CA4 of the hippocampus [6]. Pathological severity of CTE was graded using the McKee CTE staging scheme (stages I–IV, with I being least severe and IV most severe) [7]. Established criteria were used for the neuropathological diagnosis of other neurodegenerative diseases.

Independent semi-quantitative assessments of the density of p-tau pathology were performed by the aforementioned neuropathologists at the time of initial diagnosis, blinded to clinical data, using semi-quantitative rating scales (0–3 scale; 0 = none, 1 = mild, 2 = moderate, 3 = severe) in 14 regions. AT8-immunostained, 10-μm thick paraffin-embedded sections of the following regions were evaluated: dorsolateral frontal cortex, rolandic cortex, inferior frontal cortex, inferior parietal cortex, superior temporal cortex, CA1-hippocampus, CA2-hippocampus, CA4-hippocampus, entorhinal cortex, amygdala, thalamus, substantia nigra, locus coeruleus, and the dentate nucleus of the cerebellum. These regions were a priori selected because of their involvement in CTE [4–7]. The neuropathologists have very good interrater reliability for the semi-quantitative ratings of CTE stage and regional p-tau severity [7]. We created a global composite of p-tau burden based on a summary of ratings for all regions (possible range: 0–42).

#### Clinical research diagnosis of NC

For the BU ADRC, cognitive diagnoses were adjudicated during diagnostic consensus conferences that are comprised of at least one neurologist and one neuropsychologist. Consensus diagnoses were made following presentation and discussion of all examination and test findings (including review of structural MRI, if available), and social, family, and medical history. Participants who performed within the normal range (i.e., >−1.5 SD the normative mean) on all neuropsychological tests were designated as having NC. FHS screens all participants for cognitive impairment as described previously [34]. Participants were considered for this study if screening was always negative or if further assessment beyond a positive screen demonstrated NC. We included participants from the ADRC and FHS who had NC at the time closest to their MRI. This was done because it decreases likelihood of meaningful pathology at the time of MRI and because only 11 of the participants with NC were known to be deceased at the time of this study and only six of whom donated their brain and had available neuropathological diagnoses. Two had no neurodegenerative disease diagnoses, one had low AD and vascular pathological changes, one had intermediate AD pathological changes, one had primary age-related tauopathy (i.e., presence of p-tau in the medial temporal lobe in the absence of beta-amyloid plaques) [35], and one had amyotrophic lateral sclerosis (ALS). The autopsy diagnosis of ALS was based on loss of lower motor neurons in the brain stem in the context of a normal brain weight and no cortical atrophy. Based on medical record review, the MRI used for that individual was done prior to a clinical ALS diagnosis; the participant may have been manifesting early motor symptoms. Limitations associated with differences in diagnostic procedures are reviewed in the Discussion.

### Visual ratings of MRIs

We adopted previously published methods for visual rating(s) of antemortem MRIs [36]. Scans were acquired from five different manufacturers (38 GE, 33 Siemens, 13 Philips, 1 Toshiba, 1 Hitachi) that used different imaging protocols, with seven scans done on a 1.0 T, 66 on a 1.5 T, and 13 scans on a 3.0 T. Visual ratings of MRIs were performed in part due to the heterogeneity of MRI scans. Visual ratings of the scans were performed by three neuroradiologists (AM, KB, CF). At the time of the ratings, AM was (and is) an experienced attending neuroradiologist, KB was a junior attending neuroradiologist, and CF was a senior radiology resident/neuroradiology fellow. The raters were blind to the diagnostic groups, but were provided with the age at the time of scan. All images were rated in native space using RadiAnt (K.B., C.F.) or MicroDicom (A.M.) DICOM viewers. To be consistent with standard clinical reads, the raters were able to adjust contrast and zoom to their preference.

The raters used modified versions of established visual rating scales to rate patterns of atrophy, thinning, CSP, and microvascular disease. Selection of MRI features was based on the neuropathology of CTE [3, 4, 7] and those that are routinely evaluated on MRI as part of a dementia evaluation to detect and differentiate the common neurodegenerative diseases. The following were rated using T1-weighted images: orbital-frontal, dorsolateral frontal, and superior frontal lobe atrophy [37–39]; parietal-occipital lobe (i.e., posterior) atrophy [40]; anterior temporal lobe atrophy [37, 41]; MTL atrophy [42]; lateral, third, and fourth ventricular enlargement [43]; and corpus callosum thinning of the genu, body, and splenium [44]. The T1-weighted images were also used to determine the presence/absence of an anterior and posterior CSP [45]. Microvascular disease (i.e., periventricular and deep white matter hyperintensities) was rated using T2-fluid attenuated inversion recovery (FLAIR) images [46]. Susceptibility weighted images or gradient echo/T2* sequences were used to count the number of deep and lobar microbleeds. In the absence of the required sequence for a specific region, the neuroradiologist was not permitted to use other sequences to conduct ratings for that region; instead, the data were considered missing. All regions were rated on axial sequences with the exception of MTL atrophy and corpus callosum thinning, which were rated using coronal and sagittal sequences, respectively. Other orientations were also permitted to rate microbleeds in the absence of an axial in order to maximize data availability. To aid rating consistency, all scales were modified to be a five-point scale (0 = none, 1 = minimal, 2 = mild, 3 = moderate, 4 = severe), with the exception of a binary scale for anterior and posterior CSP (absent/present) and a count for microbleeds. Separate scores were provided for the left and right hemisphere, when appropriate.

The raters were trained on the visual rating scales through an iterative process. Directed readings of the visual rating scales that included reference slices and images of the targeted regions were provided to the raters and used for reference throughout the project [37–46]. The raters practiced the scales on two training data sets using images not part of the present study sample. Following completion of each practice data set, the raters met with the study behavioral neurologist (JM) and two clinical neuropsychologists (MA, RK), all of whom have expertise in neuroimaging of neurodegenerative disorders, to review their ratings and resolve discrepancies, in order to facilitate rating consistency. For analyses, the majority consensus score among the raters was used; in the absence of a majority, the median was used.

### Statistical analyses

Due to missing MRI sequences, sample sizes across the brain regions were reduced and varied (Table 1). Not all participants had T1, FLAIR, *and* SWI/GRE sequences in requisite orientations as the sequences and orientations obtained for clinical scans often vary. The sample included participants who had an available MRI regardless of the sequences present and who also met our other eligibility criteria described above. For example, there are participants who had an available axial FLAIR but no other sequences. There were also four participants who had an MRI but had none of the required sequences to perform the ratings per our methods. Interrater reliability among the three raters for each region rated on the ranked ordinal 0–4 scale was assessed using Kendall’s coefficient of concordance for ordinal variables (Kendall’s *W*). Krippendorff’s alpha was used for non-ordinal scales (i.e., absence/presence of an anterior and posterior CSP, total number of microbleeds).

**Table 1:** Visual MRI rating unadjusted means and standard deviations by region and group

	Brain donors with CTE	Normal cognition
	Mean (SD)	Mean (SD)
**Orbital-frontal (axial, 0–4 scale)**		
*N*	40	22
Left orbital-frontal	0.72 (0.78)	0.32 (0.57)
Right orbital-frontal	0.75 (0.84)	0.32 (0.57)
**Dorsolateral frontal (axial, 0–4 scale)**		
*N*	40	22
Left dorsolateral frontal	1.10 (0.93)	0.73 (0.70)
Right dorsolateral frontal	1.13 (0.91)	0.73 (0.70)
**Superior frontal (axial, 0–4 scale)**		
*N*	40	22
Left superior frontal	1.43 (0.90)	1.18 (0.80)
Right superior frontal	1.48 (0.91)	1.18 (0.80)
**Anterior temporal lobe (axial, 0–4 scale)**		
*N*	40	22
Left anterior temporal lobe	1.03 (0.89)	0.64 (0.79)
Right anterior temporal lobe	1.10 (1.06)	0.59 (0.85)
**Parietal-occipital (axial, 0–4 scale)**		
*N*	40	22
Left parietal-occipital	1.72 (1.04)	1.68 (0.89)
Right parietal-occipital	1.65 (1.00)	1.68 (0.89)
**Medial temporal lobe (coronal, 0–4 scale)**		
*N*	24	13
Left medial temporal lobe	1.08 (1.10)	0.54 (0.66)
Right medial temporal lobe	1.25 (1.03)	0.69 (0.86)
**Ventricular enlargement (axial, 0–4 scale)**		
*N*	40	22
Left lateral ventricle	1.57 (0.98)	1.09 (1.27)
Right lateral ventricle	1.55 (0.99)	1.09 (1.27)
Third ventricle	1.47 (0.99)	1.05 (1.21)
Fourth ventricle	0.03 (0.16)	0.0
**Corpus callosum (sagittal, 0–4 scale)**		
*N*	48	26
Genu of the corpus callosum	0.79 (1.03)	0.65 (0.89)
Body of the corpus callosum	1.19 (1.18)	1.04 (1.15)
Splenium of the corpus callosum	0.54 (0.82)	0.31 (0.55)
**Microvascular disease (axial, 0–4 scale)**		
*N*	43	26
Periventricular	1.86 (0.99)	1.85 (1.05)
Deep white matter	1.70 (0.96)	1.73 (0.87)
**Microbleeds,** ***n*** **(%) present**		
*N*	25	22
Deep	2 (8.0)	1 (4.5)
Frontal lobe	1 (4.0)	1 (4.5)
Temporal lobe	1 (4.0)	1 (4.5)
Parietal lobe	2 (8.0)	0
Occipital lobe	0	0
**Cavum septum pellucidum (axial)**	**N (%)**	**N (%)**
*N*	40	22
Anterior cavum septum pellucidum	13 (32.5)	2 (9.1)
Posterior cavum septum pellucidum	4 (10.0)	0

**Fig. 2:**
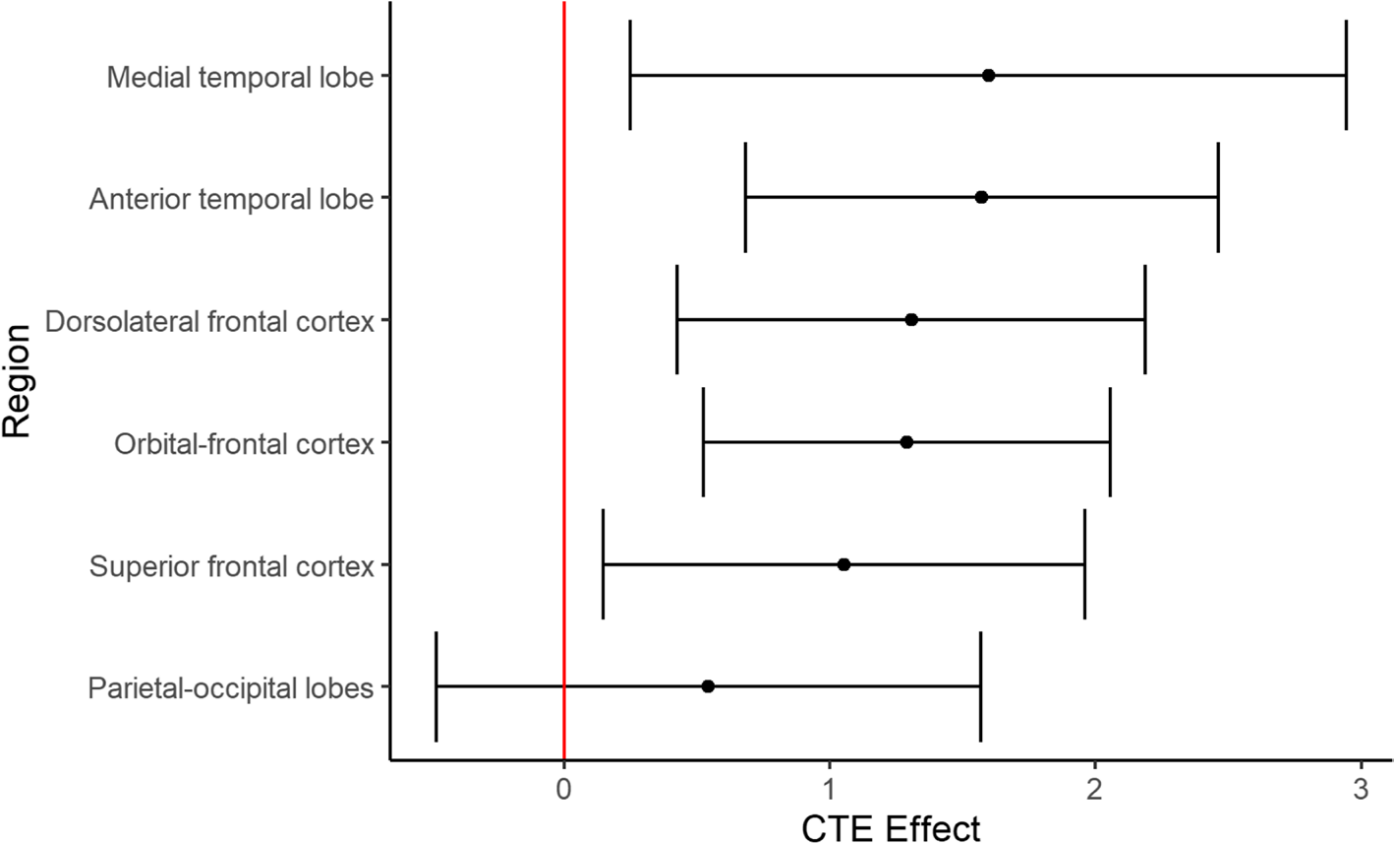
Visually rated MRI patterns of atrophy in CTE compared to participants with normal cognition. The regions (*y*-axis) were rated on a 5-point scale with 0 = none and 4 = severe and the left and right hemispheres were rated separately and combined into a summary composite for analyses (possible range: 0–8). The “Effect” represents the mean difference (black dot) between the brain donors with CTE compared to participants with normal cognition after accounting for age at the time of MRI. Higher *x*-axis scores represent higher scores (i.e., greater atrophy) in brain donors with CTE. The whiskers represent 95% confidence intervals. Statistically significant differences (i.e., false discovery rate-adjusted *p*-value less than 0.05) were found for the medial temporal lobe, anterior temporal lobe, dorsolateral frontal cortex, orbital-frontal cortex, and superior frontal cortex. There was no significant effect for the posterior-occipital lobes (*p* = 0.375).

Due to missing MRI sequences, sample sizes across the brain regions were reduced and varied. Not all participants had T1, FLAIR, *and* SWI/GRE sequences in requisite orientations as the sequences and orientations obtained for clinical scans often vary. The sample included participants who had an available MRI regardless of the sequences present and who also met our other eligibility criteria. For example, there are participants who had an available axial FLAIR but no other sequences. There were also four participants who had an MRI but had none of the required sequences to perform the ratings per our methods. Note that these are unadjusted means as compared with the adjusted mean differences in Fig. 2 and Table 2 that account for age

**Table 2:** Summary of regression models comparing brain donors with CTE and participants with normal cognition on visual rating scales

**Brain region**	**Est. marginal mean diff.**	**95% CI**	**FDR-adjusted** ***P*-value**
Orbital-frontal cortex	1.29	0.52–2.06	0.009
Dorsolateral frontal cortex	1.31	0.42–2.19	0.013
Superior frontal cortex	1.05	0.15–1.96	0.046
Anterior temporal lobes	1.57	0.68–2.46	0.009
Parietal-occipital lobes	0.54	−0.48 to 1.57	0.375
Medial temporal lobes	1.60	0.25–2.95	0.046
Lateral ventricles	1.72	0.62–2.82	0.013
Third ventricle	0.80	0.26–1.35	0.013
Fourth ventricle	0.03	−0.05 to 0.10	0.501
Corpus callosum (genu + body + splenium)	1.13	−0.13 to 2.40	0.122
Periventricular white matter hyperintensities	0.28	−0.19 to 0.74	0.330
Deep white matter hyperintensities	0.14	−0.32 to 0.59	0.553
Total number of microbleeds	0.21	−0.25 to 0.67	0.428
**Brain region**	**OR**	**95% CI**	**FDR-adjusted** ***P*-value**
Cavum septum pellucidum	6.69	1.46-50.09	0.049

The majority consensus score among the raters was used; in the absence of a majority, the median was used. Sample sizes vary across regions due to missing data as result of missing sequences from the MRI scans (see Table 1). Orbital-frontal cortex, dorsolateral frontal cortex, superior frontal cortex, anterior temporal lobes, parietal-occipital lobes, medial temporal lobes, and lateral ventricles are a summary composite of left and right hemisphere 0 (none)–4 (severe) ratings (possible range 0–8). Each region of the corpus callosum (genu, body, splenium) were separately rated on the 0–4 scale and summed. Periventricular and deep white matter hyperintensities were rated on the 0–4 scale. Absence/presence of anterior and posterior cavum septum pellucidum were rated and combined into a single variable. Total number of microbleeds is a summary composite of microbleeds in all lobes. Linear regression models were used to compare brain donors with CTE and participants with normal cognition on each visually rated region with the exception of the CSP for which binary logistic regression was used. A separate model was performed for each region and all models controlled for age at the time of the MRI scan. The estimated marginal mean differences are differences between brain donors with CTE and participants with normal cognition for the given outcome adjusted for age at MRI scan. *P*-values were false discovery rate (FDR) adjusted using the Benjamini-Hochberg Procedure

To reduce the number of analyses performed, left and right hemisphere MRI ratings (0-4 rating scale each) were combined into a single summary composite for a possible range of 0–8. For scales that had ratings for both left and right hemispheres, a symmetry model was conducted to determine whether there were statistically significant differences (using the chi-square statistic) between left and right visual rating scale scores in the donors with CTE. Summary composites of total number of microbleeds, as well as of the genu, body, and splenium of the corpus callosum were also computed. Due to the small cell sizes, anterior and posterior CSP were combined into a single binary CSP variable.

Separate linear regression models were used to compare brain donors with CTE and participants with NC on visual rating scores for all regions with the exception of the CSP. For the binary CSP outcomes, binary logistic regression was used. All effects for a given outcome were adjusted for age at MRI scan and expressed as marginal mean differences (i.e., differences in predicted values between brain donors with CTE compared to participants with NC). Two-sided statistical tests were used and *p*-values were false discovery rate adjusted using the Benjamini-Hochberg Procedure. Statistical significance was defined as a false discovery rate-adjusted alpha level less than 0.05. Given the small sample size, we place emphasis on effect sizes.

Among brain donors with CTE, multivariable linear regression analyses were conducted to examine the association between p-tau severity and atrophy on MRI. We examined the association between the global composite of p-tau severity (summary composite of semi-quantitative ratings of p-tau (possible range 0–42) across 14 cortical and subcortical brain regions, each rated on a 0 [none]–3 [severe] scale [7]) and the global composite of MRI atrophy (sum of frontal, anterior temporal, posterior, and MTL visual MRI ratings of atrophy) controlling for age at death and time since MRI. Global composites were computed and analyzed to limit the number of analyses and to increase statistical power by creating continuous scales as opposed to ordinal. Exploratory linear regression analyses examined regional correspondence between p-tau severity and the MRI ratings of atrophy. P-tau severity was assessed in the following regions that mapped onto lobes visually rated for atrophy on MRI: frontal cortex (dorsolateral frontal cortex + inferior frontal cortex), superior temporal cortex, inferior parietal cortex, and hippocampus (CA1+CA2+CA4). Note that there was missingness (*n* = 1 to 3) across the pathological variables of p-tau severity and sample sizes vary; composites were based on those with complete data across all regions.

## Results

Tables 3 and 4 present demographic and neuropathology characteristics of the sample. On average, brain donors with CTE were approximately 5 years younger at the time of the MRI scan than the participants with NC (*p*<0.01). There were no statistically significant differences between brain donors with CTE and participants with NC in terms of racial identity and years of education. Among the brain donors with CTE, the primary sport was American football for 52 of the 55 brain donors neuropathologically diagnosed with CTE (highest level played: 2 high school, 22 college, 10 semi-professional, 18 professional). Ice hockey was the primary sport for two brain donors (both played professionally) and military with combat exposure was the source of repetitive head impact exposure for one brain donor with CTE. 31.5% (17/54) served in the military, but only the one had combat exposure. 93% (50/54) were determined to have had antemortem dementia at the time of death by the diagnostic consensus panel (clinical data were not collected for one brain donor). The other four CTE brain donors had reported cognitive symptoms, but were determined to be functionally independent (i.e., not demented). The primary indication for referral for the antemortem clinical MRI for a majority of the CTE brain donors was dementia- or neurodegenerative disease-related (*n*=36; 65%). Other indications for the clinical MRI were stroke-related (*n*=8; 15%), brain tumor (*n*=5; 9%), syncope (*n*=2; 4%), seizure (*n*=2; 4%), late-onset psychotic symptoms (*n*=1; 2%), and Horner’s syndrome (*n*=1; 2%).

**Table 3:** Sample characteristics

	Brain donors with CTE	Normal Cognition	P-value^e^
*N*	55	31	--
Age at MRI scan, mean (SD) years	71.04 (7.32)	76.16 (8.55)	<0.01
Time from MRI scan to death, mean (SD) years	3.96 (3.07)	--	--
Sex, *n* (%) female	0	0	--
Race, *n* (%) White	53 (96.4)	28 (90.3)	0.35
Education, mean (SD) years^a^	16.87 (2.33)	16.17 (2.23)	0.18
Antemortem dementia, *n* (%) yes^b^	50 (92.6%)	0	--
Functional Activities Questionnaire, mean (SD)^c^	22.20 (9.02)	1.37 (3.44)	<0.01
Cause of death, *n* (%) ^d^			
Neurodegenerative disease	36 (65.5)	0	--
Cardiovascular disease	4 (7.3)	3 (27.3)	--
Suicide	2 (3.6)	0	--
Cancer	5 (9.1)	4 (36.4)	--
Motor neuron disease	3 (5.5)	1 (9.1)	--
Injury	1 (1.8)	0	--
Other/Unknown	4 (7.3)	3 (27.3)	--

^a^Education is missing for one participant with normal cognition

^b^Antemortem dementia status for the brain donors with CTE was determined by a consensus panel of clinicians based on informant-reported cognitive, behavioral, mood, and functional symptoms at time of death. Antemortem dementia was not determined for one brain donor with CTE because of missing clinical data. The four brain donors with CTE who were not determined to have had antemortem dementia did have informant-reported cognitive symptoms. The normal cognition group had normal cognition at the time of the MRI

^c^The Functional Activities Questionnaire (FAQ) assesses activities of daily living and ranges from 0 to 30 with higher scores reflecting greater functional difficulties. For brain donors with CTE, the informant of the brain donor completed the FAQ asking about difficulties at time of death. There were 6 brain donors with CTE and 12 participants with normal cognition who had missing scores for the FAQ

^d^Of the participants with normal cognition, 11 were known to be deceased and causes of death listed in the table are based on these 11 individuals. Six donated their brains for autopsy examination and two of the three other/unknown causes of death were not brain donors. Of the six brain donors, two had no neurodegenerative disease changes, one had low Alzheimer’s disease and vascular neuropathological changes, one had intermediate Alzheimer’s disease neuropathological changes, one had primary age-related tauopathy, and the other had amyotrophic lateral sclerosis. The autopsy diagnosis of amyotrophic lateral sclerosis was based on loss of lower motor neurons in the brain stem in the context of a normal brain weight and absence of cortical atrophy. Based on our medical record review, the MRI used in this study for that individual was done prior to a clinical amyotrophic lateral sclerosis diagnosis, though the participant may have been manifesting early motor symptoms

^e^Independent samples *t*-test compared brain donors with CTE to participants with normal cognition on age at MRI, years of education and FAQ scores; Fisher’s exact test was used to test for differences on race

**Table 4:** Neuropathological characteristics of brain donors with CTE

	Brain donors with CTE (***N*** = 55)		Brain donors with CTE+ (***N*** = 35)		Brain donors with CTE only (***N*** = 20)		***P***-value^a^
**Brain weight**	**Mean**	**SD**	**Mean**	**SD**	**Mean**	**SD**	**--**
Grams	1255.16	161.49	1215.60	154.66	1324.40	152.92	0.02
**Semi-quantitative ratings of p-tau severity, 0–3 scale**	**Mean**	**SD**	**Mean**	**SD**	**Mean**	**SD**	**--**
Dorsolateral frontal cortex	2.33	0.80	2.44	0.79	2.15	0.81	0.20
Rolandic cortex	1.58	1.12	1.67	1.14	1.45	1.10	0.50
Inferior frontal cortex	2.06	0.93	2.27	0.91	1.70	0.87	0.03
Inferior parietal cortex	1.98	1.02	2.09	1.04	1.79	0.98	0.31
Superior temporal cortex	2.31	0.91	2.44	0.86	2.10	0.97	0.19
CA1	1.96	1.05	2.11	1.05	1.70	1.03	0.16
CA2	1.91	1.05	1.80	1.16	2.11	0.81	0.26
CA4	1.89	1.08	1.86	1.06	1.95	1.15	0.76
Entorhinal	2.37	0.88	2.35	0.95	2.40	0.75	0.85
Amygdala	2.33	0.86	2.29	0.93	2.40	0.75	0.64
Thalamus	1.85	1.01	1.82	1.03	1.89	0.99	0.81
Substantia nigra	1.80	0.95	1.74	0.85	1.90	1.12	0.56
Locus coeruleus	2.25	0.76	2.18	0.81	2.35	0.67	0.44
Dentate nucleus	0.92	0.90	0.85	0.89	1.06	0.94	0.45
**CTE stage**	***n***	**%**	***n***	**%**	***n***	**%**	0.86
Stage I	2	3.8	1	2.9	1	5.0	
Stage II	5	9.6	4	11.4	1	5.0	
Stage III	19	34.5	12	34.3	7	35.0	
Stage IV	29	52.7	18	51.4	11	55.0	
**Alzheimer’s disease**	***n***	**%**	***n***	**%**	***n***	**%**	--
Yes	15	27.3	15	42.9	0	0	
**Lewy body disease**	***n***	**%**	***n***	**%**	***n***	**%**	--
Brain stem predominant	6	10.9	6	17.1	0	0	
Limbic (transitional)/neocortical (diffuse)	11	20.0	11	31.4	0	0	
**Frontotemporal lobar degeneration (FTLD)**	***n***	**%**	***n***	**%**	***n***	**%**	**--**
FTLD-tau	4	7.3	4	12.1	0	0	
FTLD-TDP-43	5	9.1	5	14.3	0	0	
**Motor neuron disease**	***n***	**%**	***n***	**%**	***n***	**%**	**--**
Yes	3	5.4	3	8.6	0	0	
**Prion disease**	***n***	**%**	***n***	**%**	***n***	**%**	**--**
Yes	2	3.6	2	5.7	0	0	
**CERAD neuritic plaque score**	***n***	**%**	***n***	**%**	***n***	**%**	0.03
No neuritic plaques	18	32.7	7	20.0	11	55.0	
Sparse neuritic plaques	26	47.3	18	51.4	8	40.0	
Moderate neuritic plaques	7	12.7	6	17.1	1	5.0	
Frequent neuritic plaques	4	7.3	4	11.4	0	0	
**Thal phase**	***n***	**%**	***n***	**%**	***n***	**%**	0.02
Phase 0 (A0)	5	9.1	4	11.4	1	5.0	
Phase 1/2 (A1)	8	14.5	2	5.7	6	30.0	
Phase 3 (A2)	11	20.0	5	14.3	6	30.0	
Phase 4/5 (A3)	31	56,4	24	68.6	7	35.0	
**Braak stage**	***n***	**%**	***n***	**%**	***n***	**%**	0.02
Stage 0	1	1.8	1	2.9	0	0	
Stage I/II	8	14.5	3	8.6	5	25.0	
Stage III/IV	27	49.1	14	40.0	13	65.0	
Stage V/VI	19	34.5	17	48.6	2	10.0	
**White matter rarefaction**	***n***	**%**	***n***	**%**	***n***	**%**	0.31
Moderate-severe	35	63.6	24	68.6	11	55.0	
**Arteriolosclerosis**	***n***	**%**	***n***	**%**	***n***	**%**	0.91
Moderate-severe	38	69.1	24	68.6	14	70.0	

*Note.* Brain donors with CTE+ included those with CTE and other neurodegenerative disease diagnoses whereas the CTE only group had CTE and no other neurodegenerative disease diagnoses. ^a^Independent samples *t*-test compared brain donors with CTE+ and CTE only on all variables for which mean and standard deviations are reported. Chi-square was used to test for group differences for all other variables. Due to missing data, sample sizes include *n* = 54 for dorsolateral frontal cortex, inferior parietal cortex, superior temporal cortex, CA2, and entorhinal; *n* = 53 for rolandic cortex, inferior frontal cortex, locus coeruleus, and thalamus; and *n* = 52 for dentate nucleus

The MRI indications for the NC group were diverse and related to cerebrovascular causes (*n*=7; 22.6%), memory complaints (*n*=5; 16.1%), vertigo (*n*=3; 9.7%), headaches (*n*=2; 6.5%), visual disturbances (*n*=2; 6.5%), transient global amnesia (*n*=2; 6.5%), gait instability (*n*=1; 3.2%), paresthesia (*n*=1; 3.2%), olfactory hallucinations (*n*=1; 3.2%), tinnitus (*n*=1; 3.2%), transient weakness (*n*=1; 3.2%), disequilibrium and ataxia (*n*=1; 3.2%), TBI-related (*n*=1; 3.2%), and multiple sclerosis (*n*=1; 3.2%). Two (6.5%) were done as part of participation in an outside research study. As previously mentioned, seven scans were performed on a 1.0 T, 66 on a 1.5 T, and 13 scans on a 3.0 T. Chi-square analyses showed no statistically significant differences between brain donors with CTE and participants with normal cognition on MRI magnet acquisition (*p*=0.66).

### Interrater reliability

Because there was no difference in left vs right hemisphere ratings (they were nearly identical, described below), we only examined interrater for left sided ratings. There was substantial agreement across all scales (i.e., interrater agreement statistic [Kendall’s *W* for ordinal variables, Krippendorff’s alpha for non-ordinal scales] between 0.61 and 0.80) with the exception of moderate agreement for the anterior CSP (Krippendorff’s alpha = 0.52) and fair agreement for the posterior CSP (Krippendorff’s alpha = 0.33) and fourth ventricle (Kendall’s *W* = 0.36).

### Visual MRI ratings of atrophy, CSP, and microvascular disease

Table 1 reports the means and standard deviations of all visual MRI rating scores by group. Table 2 provides a summary of the results from the multivariable linear regression models that compared brain donors with CTE and participants with NC on each visual rating scale controlling for age at the time of the MRI. Note that the “mean diff.” below refers to estimated marginal mean difference for the given outcome between the brain donors with CTE and participants with NC, adjusted for age at MRI. Findings included:

### Lobar atrophy (possible score range: 0–8)

**Fig. 3:**
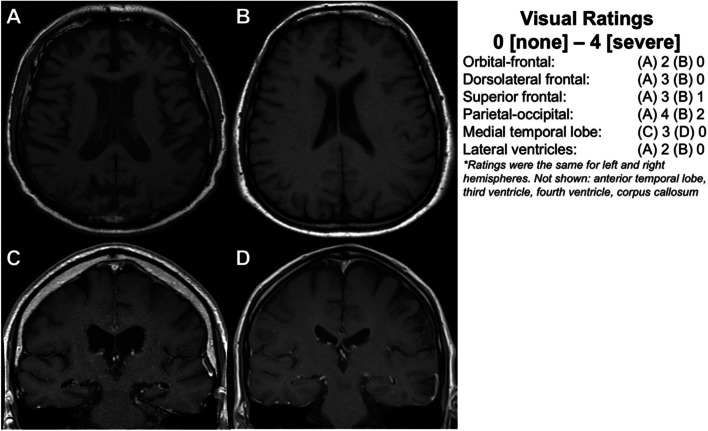
Antemortem MRI scans for brain donors with autopsy-confirmed CTE compared to participants with normal cognition. Three neuroradiologists used established visual rating scales to rate patterns of frontal, anterior temporal, parietal-occipital lobe atrophy on axial T1 sequences, as well as medial temporal lobe atrophy on coronal sequences in brain donors with CTE and participants with normal cognition. The regions were rated on a 5-point scale with 0 = none and 4 = severe. **A** Axial T1 of a male former professional American football player in his early 60’s with CTE stage IV that was rated to have mild orbital-frontal and anterior temporal lobe atrophy (not shown), moderate dorsolateral and superior frontal lobe atrophy, severe parietal-occipital lobe atrophy, and presence of an anterior and posterior cavum septum pellucidum. **B** Axial T1 of a participant with normal cognition in his late 60’s rated to have no orbital-frontal, dorsolateral frontal, or anterior temporal lobe (not shown) cortical atrophy; minimal superior frontal atrophy; mild parietal-occipital lobe atrophy; and absence of a cavum septum pellucidum. **C** and **D** are coronal sequences that show moderate hippocampal atrophy in a former professional American football player in his early 80’s with CTE stage IV (**C**) compared to no hippocampal atrophy in a participant with normal cognition in his early to mid-70s (**D**)

**Fig. 4:**
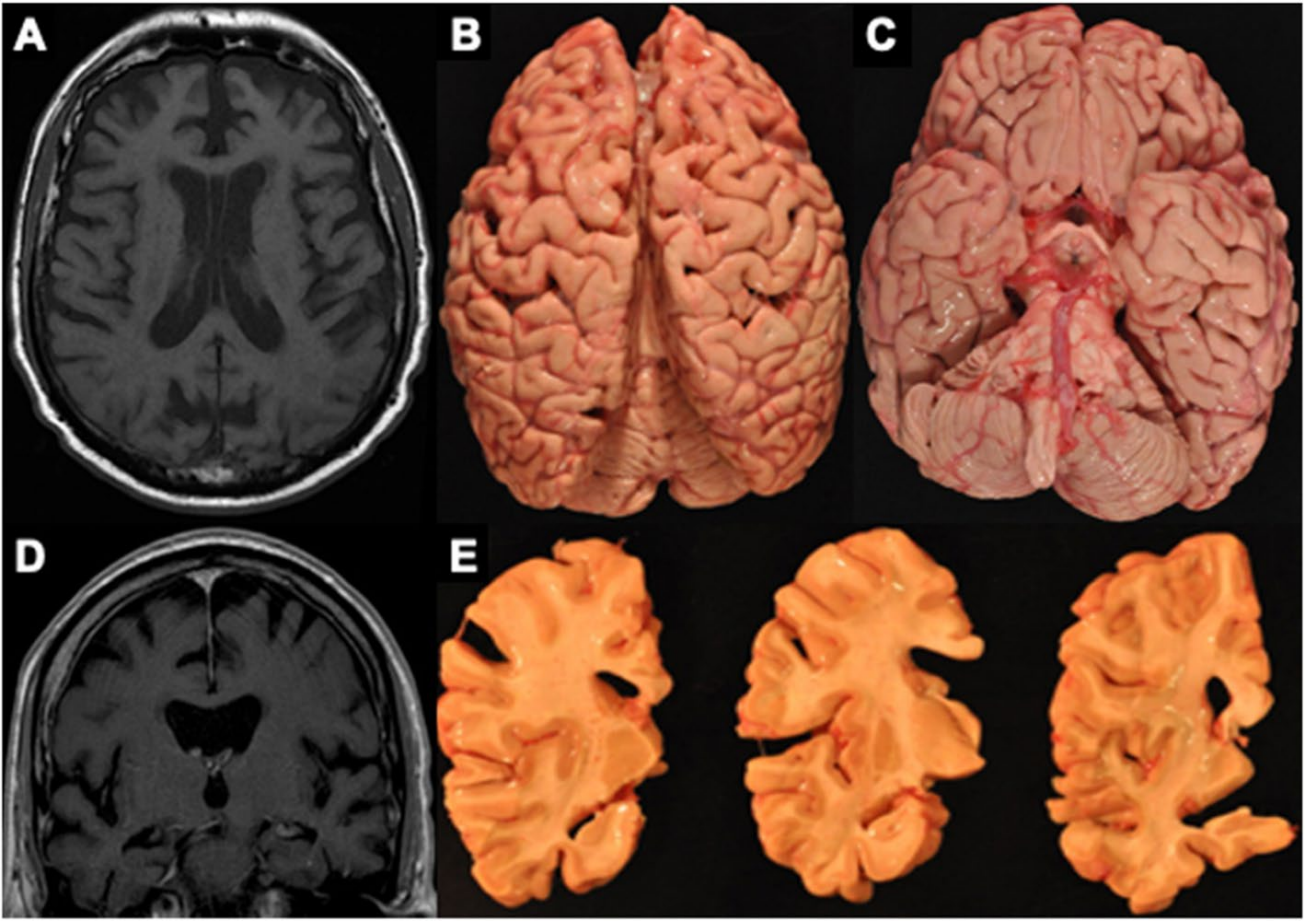
MRI and autopsy patterns of atrophy and P-tau deposition in a brain donor with CTE. The figure shows an antemortem axial (**A**) and coronal (**D**) T1 MRI sequence and corresponding gross atrophy at autopsy (**B**, **C**, **E**) of a male former professional American football player with CTE stage IV. The antemortem MRI scan was done when he was in his early 60’s and he died in his mid-70s and donated his brain. The antemortem MRI and neuropathological examination both showed frontal and temporal cortical atrophy (**A**–**E**) along with atrophy of medial temporal lobe structures (**C**, **E**) including the hippocampus and amygdala

Compared to participants with NC, brain donors with CTE had false discovery rate-corrected statistically significant higher visual MRI rating scores (i.e., greater atrophy) for the following regions: orbital-frontal cortex (mean diff.=1.29, 95% CI = 0.52–2.06, *p*=0.009), dorsolateral frontal cortex (mean diff.=1.31, 95% CI = 0.42–2.19, *p*=0.013), superior frontal cortex (mean diff.=1.05, 95% CI = 0.15–1.96, *p*=0.046), anterior temporal lobes (mean diff.=1.57, 95% CI = 0.68–2.46, *p*=0.009), and the MTL (mean diff.=1.60, 95% CI = 0.25–2.95, *p*=0.046). There were no statistically significant group differences in visual MRI ratings of parietal-occipital lobe atrophy (mean diff.=0.54, 95% CI = −0.48 to 1.57, *p*=0.375). There were no statistically significant differences in effect sizes between left and right hemisphere ratings for any of the regions in donors with CTE (*p*’s>0.10 for all). Figure 2 displays group differences in regional atrophy ratings in order of effect size magnitude. Figure 3 shows an exemplar MRI scan of brain donors with CTE and participants with NC. Figure 4 shows an antemortem MRI scan and gross brain photographs taken at autopsy for a brain donor with CTE.

#### Ventricle size

Brain donors with CTE had statistically significant higher visual MRI rating scores (i.e., greater ventricle size) for the lateral ventricles (mean diff.=1.72 [0–8 scale], 95% CI = 0.62–2.82, *p*=0.013) and the third ventricle (mean diff.=0.80 [0–4 scale], 95% CI=0.26–1.35, *p*=0.013). See Fig. 3 as an example. There were no statistically significant differences in effect sizes between ratings of the left and right lateral ventricles (*p*=1.00). There was not a statistically significant group difference on visual MRI ratings of the fourth ventricle (*p*=0.501).

#### Cavum septum pellucidum (absence/presence)

Thirteen brain donors with CTE (33%) had an anterior CSP, whereas 2 of the participants with NC (9%) had an anterior CSP (Fig. 3). Four brain donors with CTE had a posterior CSP, whereas none of the participants with NC had a posterior CSP. Three of the brain donors with CTE had both an anterior and posterior CSP. Overall, the brain donors with CTE were at 6.7X (95% CI = 1.5–50.1, *p*=0.049) increased odds for having a CSP.

#### Corpus callosum thinning

There was not a statistically significantly group effect for thinning of the corpus callosum (*p*>0.05).

#### Microvascular disease

There were no statistically significant group differences on FLAIR MRI ratings for periventricular WMH (*p*=0.330), deep WMH (*p*=0.553), or number of microbleeds (*p*=0.428).

### Sensitivity analyses: excluding co-morbidities

Thirty-five (63.6%) of the brain donors with CTE had a co-morbid neurodegenerative disease diagnosis (i.e., CTE+) and 20 had CTE and no other neurodegenerative disease diagnoses. Of the brain donors with CTE (*n*=55), 9 (16.4%) had FTLD (*n*=4 tau, *n*=5 TDP-43). Given the frontal-temporal similarities in atrophy between CTE and frontotemporal lobar degeneration, we repeated the linear regression models for the frontal (i.e., orbital-frontal, dorsolateral frontal and superior frontal cortex) and anterior temporal lobes after excluding CTE brain donors who had a co-morbid frontotemporal lobar degeneration neuropathological diagnosis (CTE+FTLD). Effect sizes remained similar (i.e., less than 10% change in estimates). In addition, 15 (27.3%) had a neuropathological diagnosis of AD. Although we did not observe group differences on visual ratings of posterior atrophy (as would be expected with AD), there was a group effect for the MTL—a hallmark MRI finding in AD. We repeated the model with MTL as the outcome after excluding CTE brain donors with AD (CTE+AD). There was a small 11.3% *increase* in the effect size estimate (mean diff.=1.78, 95% CI = 0.43–3.13, *p*=0.01), suggesting observed differences were not explained by AD pathology. Effect sizes remained similar for atrophy and ventricle size when the two brain donors with prion disease and the three with motor neuron disease were excluded (5% or less change in effect size with the exception of a 10.4% and 10.8% *increase* in effect for the superior frontal and MTL, respectively, when prion disease was excluded). As shown in Table 4, independent samples *t*-tests and chi-square analyses showed no statistically significant differences between the brain donors with CTE+ and CTE only on the semi-quantitative rating scales of p-tau severity across 14 cortical and subcortical brain regions (except the inferior frontal cortex), semi-quantitative ratings of atrophy at autopsy (except the right frontal lobe), CTE stage, white matter rarefaction, or arteriolosclerosis.

### P-Tau severity and MRI atrophy in brain donors with CTE

We examined the association between p-tau severity (rated at autopsy by neuropathologists who were blinded to clinical data and MRI ratings) and visually rated MRI atrophy (Table 5). Linear regressions showed that the global visual MRI atrophy composite was associated with the global summary composite of p-tau (standardized beta=0.68, SE=0.22, *p*<0.01) controlling for age at death and time from MRI to death. Greater total p-tau burden was associated with greater total brain atrophy on MRI. As sensitivity analyses, we repeated the above linear regression models after first excluding brain donors with CTE+FTLD, followed by excluding those with CTE+AD. The association between the global visual MRI atrophy composite and the global summary composite of p-tau remained after exclusion of brain donors with CTE+AD (standardized beta=0.84, SE=0.19, *p*<0.01) and CTE+FTLD (standardized beta=0.60, SE=0.21, *p*=0.01).

**Table 5:** Summary of regression models examining the effect of P-tau severity on atrophy in the brain donors with CTE

	Effects of p-tau severity on atrophy		
Measures of atrophy	Standardized beta	Standard error	***P***-value
^a^**Visually rated MRI global atrophy**	0.68	0.22	<0.01
Frontal lobes	0.30	0.15	0.047
Temporal lobes	0.23	0.15	0.15
Posterior lobes	0.08	0.15	0.62
Medial temporal lobes	0.25	0.21	0.25

A summary composite of semi-quantitative ratings of p-tau (0 [none]–3 [severe] scale) across 14 cortical and subcortical brain regions was computed^7^ and served as the independent variable. A global composite of MRI atrophy was calculated, based on the sum of frontal (orbital-frontal cortex, dorsolateral frontal cortex, superior frontal cortex), anterior temporal, posterior (parietal, occipital), and MTL visual MRI ratings of atrophy. P-tau severity was assessed in the following regions that mapped onto lobes visually rated for atrophy on MRI: frontal cortex (dorsolateral frontal cortex + inferior frontal cortex), superior temporal cortex, inferior parietal cortex, and hippocampus (CA1+CA2+CA4)

^a^The primary analyses included linear regression models that tested the association between global-based composites to limit the number of analyses and to increase statistical power. Exploratory linear regression analyses examined regional correspondence between p-tau severity and the MRI ratings of atrophy. Analyses controlled for age at death and time since MRI

Exploratory linear regression analyses showed regional correspondence between frontal p-tau severity and frontal MRI atrophy (orbital-frontal + dorsolateral frontal + superior frontal) (standardized beta=0.30, SE=0.15, *p*=0.047). Greater frontal p-tau severity correlated with greater frontal atrophy on MRI. There were no other statistically significant regional effects between p-tau severity and atrophy on MRI.

## Discussion

Based on visual ratings of antemortem MRIs obtained from medical record requests, brain donors who had autopsy-confirmed CTE had more severe frontal and anterior temporal lobe atrophy, MTL atrophy, lateral and third ventricular enlargement, and increased odds for having a CSP compared to participants with NC. There was no evidence of left vs right asymmetry in atrophy. There were no statistically significant differences in ratings of posterior atrophy (i.e., parietal-occipital lobes) or microvascular disease between brain donors with CTE and participants with NC. Additionally, we found that more severe p-tau pathology (rated at autopsy) was associated with greater MRI ratings of atrophy among the brain donors with CTE. In summary, the current findings provide, for the first time, insight into the structural MRI profiles of people with neuropathologically confirmed CTE, as well as support p-tau accumulation as a correlate of atrophy in CTE.

Compared to participants with NC, brain donors with CTE had greater visually rated frontal-temporal and MTL atrophy on MRI. These findings match the neuroanatomical distribution of CTE p-tau pathology and the corresponding gross neuropathology [3, 4, 6, 7]. In CTE, there is early p-tau involvement in the dorsolateral frontal cortices [3, 4, 6, 7], the region of the frontal lobe for which we found the largest group differences. The hippocampus is also markedly affected in CTE, but later in the disease course and with CA2 and CA4 hippocampal subfields disproportionately affected [6, 7]. Third ventricle enlargement may also result from thalamic atrophy in CTE [3, 4]. Our findings are also consistent with in vivo studies that show tau positron emission tomography (flortaucipir) frontal-temporal and MTL binding [8, 9], as well as reduced MRI-derived volumetrics of frontal and temporal lobes [9] and MTL structures (e.g., amygdala, hippocampus) [9, 11–14] among people at high risk for having CTE neuropathology (e.g., former National Football League players, professional fighters). Taken together, there is converging evidence for frontotemporal and MTL atrophy in CTE that might be able to be visualized on MRI.

Our observed effect estimates remained similar when donors with CTE+AD and CTE+FTLD pathological diagnoses were excluded. Neuropathologically, CTE is distinguished from AD by the lack of beta amyloid neuritic plaques and a distinctive pattern, type, and regional distribution of p-tau pathology [6, 8, 47]. Although the present findings provide insight into potential MRI differential diagnostic patterns for CTE, inferences on disease specific differences are limited due to lack of an AD, FTLD, or other neurodegenerative disease comparison groups. The use of a NC group allows for testing of the usefulness of the biomarker for disease detection; if the biomarker cannot discriminate from NC (other limitations notwithstanding) it is unlikely to be a useful biomarker. Specificity of our findings remain unknown without a neurodegenerative disease comparison group and future research is underway to address this knowledge gap.

We found that brain donors with CTE were at 6.7X increased odds for a CSP compared to participants with NC. In autopsy-confirmed CTE, there are frequently abnormalities of the septum pellucidum [3, 4, 6]. The in vivo MRI literature shows an association between exposure to repetitive head impacts and the presence of a CSP [9, 17, 18, 48, 49]. However, a CSP is not specific to CTE and is a frequent MRI finding in the general adult population. A CSP may not be associated with specific clinical symptoms and may be better viewed as a marker of global injury associated with repetitive head impact exposure. Regardless, the presence of a CSP may be a supportive differential diagnostic feature for CTE when combined with other relevant risk factor, clinical, and neuroimaging data points. There are challenges for the accurate and reliable detection of a CSP, as evidenced by the fair to moderate interrater agreement among the neuroradiologists. A small CSP can easily be missed and/or a posterior CSP can be mistaken for detachment of the fornix.

In other tauopathies, such as AD and FTLD-tau, p-tau pathology is a contributor to neurodegeneration [19–21]. Although p-tau may induce microtubule disruption, protein aggregation, and alterations in protein expression, the exact mechanism by which p-tau triggers neurodegeneration is unclear. The neurodegeneration in CTE has similarly been hypothesized to be a result of CTE p-tau pathology. This study provides empirical support for this hypothesis by showing an association between p-tau severity and atrophy on antemortem MRI. This association remained after excluding donors who had co-morbid AD and FTLD, suggesting unique contribution from CTE and this is consistent with research showing the molecular composition of p-tau is distinct from AD and FTLD [47, 50, 51]. Similar to other neurodegenerative diseases, widespread p-tau pathology in CTE is likely needed for neurodegeneration to result in clinical syndromes (e.g., dementia) [28].

### Limitations

The study has limitations. The restricted and variable sample sizes across sequences due to missingness are important limitations of this study and effect estimates and p-values should be interpreted together and with caution. Our findings best generalize to a clinic-based population (i.e., those who present for a dementia evaluation and undergo MRI). The external validity is limited by potential selection biases associated with brain donation. Our previous data show that brain donation selection biases do not invalidate exposure to repetitive head impact-CTE associations [5]. We would not expect observed atrophy and tau relationships to be different in people with CTE who did not donate their brains, a requirement for brain bank selection to bias the current findings. We accounted for differences in the time interval between antemortem MRI and death in our models, but this remains a limitation because the presence or severity of pathology at the time of MRI was unknown. It would have been optimal to examine associations between visual ratings of MRI atrophy and metrics of atrophy at autopsy. However, current metrics of pathological atrophy are relatively crude (e.g., semi-quantitative scales) and issues with measurement and other reasons (e.g., statistical power) are unable to be overcome without a larger sample size and more refined measures of atrophy. The visual ratings of atrophy in the brain donors with CTE in this sample tended to be of mild severity on average and the clinical significance of both the MRI atrophy and pathology at autopsy require further investigation. Given 93% of brain donors with CTE were judged to have had antemortem dementia, we were unable to examine dementia as an outcome. Future research is currently underway to examine the various cognitive, mood, and behavioral symptoms associated with p-tau in CTE.

There are limitations of the NC group. The neuropathological status of the NC group was largely unknown, with only six coming to autopsy. Given the absence of cognitive impairment, we assumed the NC group does not have meaningful neuropathology. Given there were participants from the NC group who came to autopsy and had pathological evidence of neurodegenerative disease, this may not be the case. The presence of pathology in the NC group would likely bias results towards the null and the reported effects might be an underestimate. The sample was restricted to 60 years or older to have a similar age distribution between the CTE and NC groups. Even after this restriction, participants with NC were on average approx. 5 years older and this group difference might have underestimated the effects. Lastly, the sample size was small, particularly of the NC group, and the null effects (e.g., for posterior atrophy) should be interpreted with caution.

We obtained antemortem MRIs via medical record request resulting in scans that were heterogenous in terms of quality and acquisition parameters (e.g., resolution, slice thickness, scanner type). For these reasons, we used established visual rating methods as opposed to automated software (e.g., FreeSurfer). Visual ratings have ecological validity and the use of established rating scales allows for replication. The scales are subjective and can introduce measurement error. The three trained neuroradiologists had relatively good agreement on the visual rating scales, which is consistent with other imaging-pathological studies [36]. It is reassuring that previous in vivo research using research grade MRIs and automated image analysis software in samples at high risk for CTE found similar patterns [9, 11–14, 17, 18, 48, 49]. Prospective clinical-imaging-pathological correlation studies are the gold standard and will be essential to validate the current findings. Such studies are ongoing [8, 9], but it will require years to gather sufficient clinicopathological data for meaningful analysis. There is a timely need to identify potential biomarker targets that can be used in conjunction with current research diagnostic criteria for CTE (i.e., traumatic encephalopathy syndrome) to support a diagnosis of “probable CTE” [52, 53].

## Conclusions

Cognitively symptomatic male brain donors with autopsy-confirmed CTE had more severe visually rated frontal, temporal, and hippocampal atrophy and increased odds for having a CSP on antemortem MRI scans compared to same-age men with NC. In addition, more severe p-tau pathology was associated with greater MRI ratings of atrophy. If validated with prospective clinical-pathological correlation studies, these findings support the use of structural MRI as a valuable tool to support a diagnosis of CTE during life.

## Data Availability

Most data associated with this study are available in the main text. UNITE data is also made available through FITBIR. Individual MRI scans are not made freely available to protect privacy. However, MRI data will be made available in FITBIR in the future and this data and other data can also be shared upon reasonable request.

## Abbreviations

AD: Alzheimer’s disease
ALS: Amyotrophic lateral sclerosis
BU ADRC: Boston University Alzheimer’s Disease Research Center
CSP: Cavum septum pellucidum
CTE: Chronic traumatic encephalopathy
FHS: Framingham Heart Study
FLAIR: Fluid attenuated inversion recovery
FTD: Frontotemporal dementia
FTLD: Frontotemporal lobar degeneration
MRI: Magnetic resonance imaging
MTL: Medial temporal lobe
NC: Normal cognition
RHI: Repetitive head impacts
SWI: Susceptibility weighted imaging
UNITE: Understanding Neurologic Injury in Traumatic Encephalopathy
P-tau: Hyper-phosphorylated tau
